# 3-(1,3-Benzodioxol-5-yl)-3*H*-benzo[*f*]isobenzofuran-1-one

**DOI:** 10.1107/S1600536810010068

**Published:** 2010-03-20

**Authors:** S. Thenmozhi, A. SubbiahPandi, J. Arulclement, A. K. MohanaKrishnan

**Affiliations:** a Department of Physics, Presidency College (Autonomous), Chennai 600 005, India; b Department of Organic Chemistry, University of Madras, Guindy Campus, Chennai 600 025, India

## Abstract

In the title compound, C_19_H_12_O_4_, the dioxole ring adopts a flattened envelope conformation with the methyl­ene C at the flap [deviation = 0.104 (2) Å]. The benzene ring of the benzodioxole ring system makes a dihedral angle of 76.45 (5)° with the planar [maximum deviation = 0.016 (1) Å] 3*H*-benzo[*f*]isobenzofuran-1-one ring system. In the crystal structure, the mol­ecules are linked into *C*(5) chains running along the *b* axis by inter­molecular C—H⋯O hydrogen bonds. In addition, C—H⋯π inter­actions are observed.

## Related literature

For the biological activity of benzofuran compounds, see: Howlett *et al.* (1999 ▶); Twyman & Allsop (1999 ▶); Valerga *et al.* (2009 ▶).
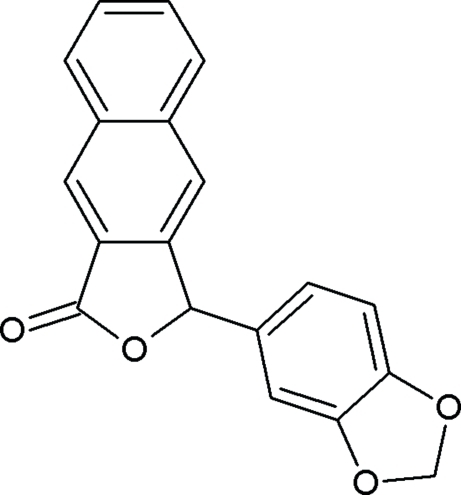


## Experimental

### 

#### Crystal data

C_19_H_12_O_4_
*M* *_r_* = 304.29
Monoclinic, 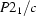

*a* = 7.8617 (3) Å
*b* = 12.0417 (4) Å
*c* = 15.0214 (5) Å
β = 95.848 (2)°
*V* = 1414.65 (9) Å^3^
*Z* = 4
Mo *K*α radiation
μ = 0.10 mm^−1^
*T* = 293 K
0.21 × 0.19 × 0.17 mm

#### Data collection

Bruker Kappa APEXII CCD diffractometer
Absorption correction: multi-scan (*SADABS*; Sheldrick, 1996 ▶) *T*_min_ = 0.979, *T*_max_ = 0.983
19039 measured reflections
4251 independent reflections
3370 reflections with *I* > 2σ(*I*)
*R*_int_ = 0.038

#### Refinement

*R*[*F* ^2^ > 2σ(*F* ^2^)] = 0.046
*wR*(*F* ^2^) = 0.137
*S* = 1.02
4251 reflections
208 parameters
H-atom parameters constrained
Δρ_max_ = 0.34 e Å^−3^
Δρ_min_ = −0.22 e Å^−3^

### 

Data collection: *APEX2* (Bruker, 2004 ▶); cell refinement: *SAINT* (Bruker, 2004 ▶); data reduction: *SAINT*; program(s) used to solve structure: *SHELXS97* (Sheldrick, 2008 ▶); program(s) used to refine structure: *SHELXL97* (Sheldrick, 2008 ▶); molecular graphics: *ORTEP-3* (Farrugia, 1997 ▶); software used to prepare material for publication: *SHELXL97* and *PLATON* (Spek, 2009 ▶).

## Supplementary Material

Crystal structure: contains datablocks global, I. DOI: 10.1107/S1600536810010068/ci5047sup1.cif

Structure factors: contains datablocks I. DOI: 10.1107/S1600536810010068/ci5047Isup2.hkl

Additional supplementary materials:  crystallographic information; 3D view; checkCIF report

## Figures and Tables

**Table 1:** Hydrogen-bond geometry (Å, °) *Cg*1 is the centroid of the C13–C18 ring.

*D*—H⋯*A*	*D*—H	H⋯*A*	*D*⋯*A*	*D*—H⋯*A*
C12—H12⋯O1^i^	0.98	2.39	3.3277 (16)	159
C3—H3⋯*Cg*1^ii^	0.93	2.84	3.6021 (15)	140

Symmetry codes: (i) 
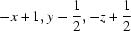
; (ii) 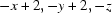.

**Fig. 1.**
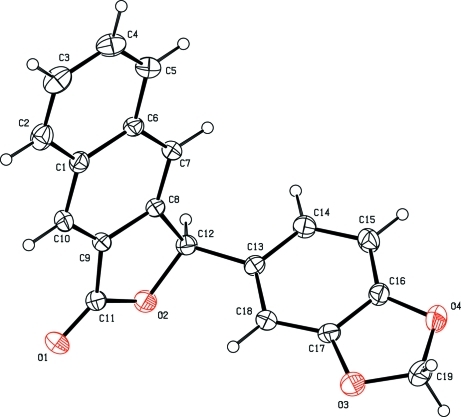
The molecular structure of the title compound, showing the atom-numbering scheme. Displacement ellipsoids are drawn at the 30% probability level.

## supplementary crystallographic information

### Comment

Molecules containing a benzofuran ring system have attracted considerable interest in view of their biological and pharmacological properties (Howlett *et al.*, 1999; Twyman & Allsop, 1999). Furan compounds exhibit antibacterial and antifungal activities (Valerga *et al.*, 2009). Against this background and in order to obtain detailed information on molecular conformation in the solid state, an X-ray crystallographic study of the title compound has been carried out and the results are presented here.

The 3H-benzo[f]isobenzofuran-1-one ring system is essentially planar, with a maximum deviation of 0.016 (1) Å for atom C12. In the 1,3-benzodioxole ring system, the dioxole ring adopts a flattened envelope conformation with the methylene C at the flap [deviation = 0.104 (2) Å]. The benzene ring of the benzodioxole ring system makes a dihedral angle of 76.45 (5)° with the 3H-benzo[f]isobenzofuran-1-one ring system.

In the crystal structure, intermolecular C—H···O hydrogen bonds (Table 1) involving atoms C12 and O1, link the molecules into chains which run parallel to the *b* axis and can be described by a graph set motif of C(5). In addition, the crystal packing is stabilized by C—H···π interactions involving the C13–C18 ring.

### Experimental

NaBH_4_ (1.6 g, 43.75 mmol) was carefully added in small portions to a solution of keto acid (3.5 g, 10.93 mmol) in THF-EtOH (2:5) at 273 K. The reaction mixture was refluxed for 12 h and then poured into ice water (200 ml). The reaction mixture was acidified using HCl (pH = 2-3) and then stirred for 0.5 h at room temperature. The solid formed was filtered and washed with methanol to afford lactone as a colourless solid.

### Refinement

H atoms were positioned geometrically and allowed to ride on their parent C atoms, with C–H distances fixed in the range 0.93–0.98 Å and U_iso_(H) = 1.2U_eq_(C).

### Crystal data

**Table:**

C_19_H_12_O_4_	*F*(000) = 632
*M**_r_* = 304.29	*D*_x_ = 1.429 Mg m^−^^3^
Monoclinic, *P*2_1_/*c*	Mo *K*α radiation, λ = 0.71073 Å
Hall symbol: -P 2ybc	Cell parameters from 4251 reflections
*a* = 7.8617 (3) Å	θ = 2.2–30.4°
*b* = 12.0417 (4) Å	µ = 0.10 mm^−^^1^
*c* = 15.0214 (5) Å	*T* = 293 K
β = 95.848 (2)°	Block, colourless
*V* = 1414.65 (9) Å^3^	0.21 × 0.19 × 0.17 mm
*Z* = 4	

### Data collection

**Table:**

Bruker Kappa APEXII CCD diffractometer	4251 independent reflections
Radiation source: fine-focus sealed tube	3370 reflections with *I* > 2σ(*I*)
graphite	*R*_int_ = 0.038
ω scans	θ_max_ = 30.4°, θ_min_ = 2.2°
Absorption correction: multi-scan (*SADABS*; Sheldrick, 1996)	*h* = −11→10
*T*_min_ = 0.979, *T*_max_ = 0.983	*k* = −17→16
19039 measured reflections	*l* = −21→13

### Refinement

**Table:**

Refinement on *F*^2^	Primary atom site location: structure-invariant direct methods
Least-squares matrix: full	Secondary atom site location: difference Fourier map
*R*[*F*^2^ > 2σ(*F*^2^)] = 0.046	Hydrogen site location: inferred from neighbouring sites
*wR*(*F*^2^) = 0.137	H-atom parameters constrained
*S* = 1.02	*w* = 1/[σ^2^(*F*_o_^2^) + (0.0727*P*)^2^ + 0.2801*P*] where *P* = (*F*_o_^2^ + 2*F*_c_^2^)/3
4251 reflections	(Δ/σ)_max_ = 0.002
208 parameters	Δρ_max_ = 0.34 e Å^−^^3^
0 restraints	Δρ_min_ = −0.22 e Å^−^^3^

### Special details

**Table:**

Geometry. All esds (except the esd in the dihedral angle between two l.s. planes) are estimated using the full covariance matrix. The cell esds are taken into account individually in the estimation of esds in distances, angles and torsion angles; correlations between esds in cell parameters are only used when they are defined by crystal symmetry. An approximate (isotropic) treatment of cell esds is used for estimating esds involving l.s. planes.
Refinement. Refinement of *F*^2^ against ALL reflections. The weighted *R*-factor wR and goodness of fit *S* are based on *F*^2^, conventional *R*-factors *R* are based on *F*, with *F* set to zero for negative *F*^2^. The threshold expression of *F*^2^ > σ(*F*^2^) is used only for calculating *R*-factors(gt) etc. and is not relevant to the choice of reflections for refinement. *R*-factors based on *F*^2^ are statistically about twice as large as those based on *F*, and *R*- factors based on ALL data will be even larger.

### Fractional atomic coordinates and isotropic or equivalent isotropic displacement parameters (Å^2^)

**Table:**

	*x*	*y*	*z*	*U*_iso_*/*U*_eq_	
O1	0.50740 (15)	1.28238 (9)	0.19129 (8)	0.0587 (3)	
O2	0.58783 (11)	1.12957 (8)	0.26946 (6)	0.0419 (2)	
O3	1.20824 (14)	1.15928 (9)	0.41662 (8)	0.0588 (3)	
O4	1.29651 (13)	0.98350 (9)	0.46223 (8)	0.0548 (3)	
C1	0.70723 (14)	1.07497 (10)	−0.02366 (7)	0.0333 (2)	
C2	0.71799 (18)	1.09700 (12)	−0.11570 (9)	0.0443 (3)	
H2	0.6798	1.1648	−0.1397	0.053*	
C3	0.7832 (2)	1.02046 (14)	−0.16932 (9)	0.0503 (4)	
H3	0.7902	1.0364	−0.2294	0.060*	
C4	0.84016 (19)	0.91727 (13)	−0.13420 (9)	0.0479 (3)	
H4	0.8842	0.8652	−0.1714	0.057*	
C5	0.83164 (17)	0.89258 (11)	−0.04622 (9)	0.0401 (3)	
H5	0.8700	0.8239	−0.0241	0.048*	
C6	0.76513 (14)	0.97022 (9)	0.01198 (7)	0.0307 (2)	
C7	0.75593 (14)	0.94554 (9)	0.10366 (7)	0.0313 (2)	
H7	0.7938	0.8775	0.1274	0.038*	
C8	0.69034 (13)	1.02365 (9)	0.15634 (7)	0.0289 (2)	
C9	0.63280 (14)	1.12596 (9)	0.12091 (8)	0.0324 (2)	
C10	0.64042 (16)	1.15336 (10)	0.03305 (8)	0.0369 (3)	
H10	0.6023	1.2222	0.0112	0.044*	
C11	0.56804 (15)	1.19097 (11)	0.19281 (9)	0.0396 (3)	
C12	0.66602 (15)	1.02200 (10)	0.25451 (7)	0.0329 (2)	
H12	0.5871	0.9622	0.2667	0.039*	
C13	0.83094 (15)	1.00982 (10)	0.31446 (7)	0.0310 (2)	
C14	0.88263 (17)	0.90475 (10)	0.34263 (9)	0.0389 (3)	
H14	0.8110	0.8446	0.3280	0.047*	
C15	1.04087 (17)	0.88686 (11)	0.39288 (9)	0.0420 (3)	
H15	1.0765	0.8161	0.4114	0.050*	
C16	1.13967 (16)	0.97803 (11)	0.41324 (8)	0.0364 (3)	
C17	1.08753 (16)	1.08282 (10)	0.38590 (8)	0.0355 (2)	
C18	0.93465 (15)	1.10207 (10)	0.33622 (8)	0.0351 (2)	
H18	0.9013	1.1732	0.3178	0.042*	
C19	1.34790 (18)	1.09710 (13)	0.45929 (10)	0.0497 (3)	
H19A	1.3783	1.1248	0.5195	0.060*	
H19B	1.4468	1.1041	0.4260	0.060*	

### Atomic displacement parameters (Å^2^)

**Table:**

	*U*^11^	*U*^22^	*U*^33^	*U*^12^	*U*^13^	*U*^23^
O1	0.0624 (7)	0.0454 (6)	0.0653 (7)	0.0244 (5)	−0.0085 (5)	−0.0180 (5)
O2	0.0383 (5)	0.0475 (5)	0.0398 (4)	0.0108 (4)	0.0030 (4)	−0.0106 (4)
O3	0.0477 (6)	0.0393 (5)	0.0841 (8)	−0.0062 (4)	−0.0198 (5)	−0.0088 (5)
O4	0.0438 (5)	0.0521 (6)	0.0637 (7)	0.0030 (4)	−0.0172 (5)	0.0006 (5)
C1	0.0314 (5)	0.0338 (6)	0.0336 (5)	−0.0029 (4)	−0.0020 (4)	0.0019 (4)
C2	0.0467 (7)	0.0483 (7)	0.0367 (6)	−0.0045 (6)	−0.0013 (5)	0.0095 (5)
C3	0.0534 (8)	0.0644 (9)	0.0342 (6)	−0.0127 (7)	0.0097 (5)	0.0010 (6)
C4	0.0511 (8)	0.0527 (8)	0.0424 (7)	−0.0085 (6)	0.0165 (6)	−0.0114 (6)
C5	0.0432 (6)	0.0367 (6)	0.0415 (6)	−0.0020 (5)	0.0094 (5)	−0.0059 (5)
C6	0.0292 (5)	0.0291 (5)	0.0335 (5)	−0.0028 (4)	0.0014 (4)	−0.0027 (4)
C7	0.0343 (5)	0.0248 (5)	0.0342 (5)	0.0013 (4)	0.0011 (4)	−0.0002 (4)
C8	0.0271 (5)	0.0266 (5)	0.0322 (5)	−0.0010 (4)	−0.0010 (4)	−0.0023 (4)
C9	0.0306 (5)	0.0267 (5)	0.0385 (5)	0.0038 (4)	−0.0037 (4)	−0.0043 (4)
C10	0.0391 (6)	0.0283 (5)	0.0412 (6)	0.0044 (4)	−0.0056 (5)	0.0026 (4)
C11	0.0333 (6)	0.0383 (6)	0.0453 (6)	0.0072 (5)	−0.0056 (5)	−0.0111 (5)
C12	0.0319 (5)	0.0336 (6)	0.0331 (5)	0.0005 (4)	0.0026 (4)	−0.0039 (4)
C13	0.0328 (5)	0.0335 (5)	0.0270 (5)	−0.0011 (4)	0.0037 (4)	−0.0009 (4)
C14	0.0402 (6)	0.0319 (6)	0.0440 (6)	−0.0052 (5)	0.0016 (5)	0.0033 (5)
C15	0.0453 (7)	0.0334 (6)	0.0463 (7)	0.0007 (5)	−0.0004 (5)	0.0083 (5)
C16	0.0364 (6)	0.0401 (6)	0.0320 (5)	0.0031 (5)	0.0000 (4)	0.0004 (4)
C17	0.0372 (6)	0.0325 (6)	0.0363 (5)	−0.0029 (4)	0.0010 (4)	−0.0061 (4)
C18	0.0385 (6)	0.0290 (5)	0.0373 (5)	0.0011 (4)	0.0005 (4)	−0.0013 (4)
C19	0.0391 (7)	0.0562 (9)	0.0515 (8)	−0.0040 (6)	−0.0063 (6)	−0.0052 (6)

### Geometric parameters (Å, °)

**Table:**

O1—C11	1.1988 (15)	C7—H7	0.93
O2—C11	1.3639 (17)	C8—C9	1.3988 (15)
O2—C12	1.4610 (14)	C8—C12	1.5063 (15)
O3—C17	1.3682 (15)	C9—C10	1.3675 (17)
O3—C19	1.4260 (18)	C9—C11	1.4665 (16)
O4—C16	1.3716 (15)	C10—H10	0.93
O4—C19	1.4283 (18)	C12—C13	1.5085 (16)
C1—C10	1.4085 (17)	C12—H12	0.98
C1—C2	1.4188 (17)	C13—C14	1.3820 (17)
C1—C6	1.4266 (16)	C13—C18	1.3969 (16)
C2—C3	1.358 (2)	C14—C15	1.4044 (18)
C2—H2	0.93	C14—H14	0.93
C3—C4	1.405 (2)	C15—C16	1.3615 (18)
C3—H3	0.93	C15—H15	0.93
C4—C5	1.3628 (19)	C16—C17	1.3765 (17)
C4—H4	0.93	C17—C18	1.3684 (17)
C5—C6	1.4160 (16)	C18—H18	0.93
C5—H5	0.93	C19—H19A	0.97
C6—C7	1.4178 (15)	C19—H19B	0.97
C7—C8	1.3638 (15)		
			
C11—O2—C12	111.37 (9)	O1—C11—O2	121.65 (12)
C17—O3—C19	105.91 (11)	O1—C11—C9	130.04 (13)
C16—O4—C19	105.73 (10)	O2—C11—C9	108.31 (10)
C10—C1—C2	121.80 (11)	O2—C12—C8	103.66 (9)
C10—C1—C6	119.34 (10)	O2—C12—C13	110.11 (9)
C2—C1—C6	118.86 (11)	C8—C12—C13	113.49 (9)
C3—C2—C1	120.96 (13)	O2—C12—H12	109.8
C3—C2—H2	119.5	C8—C12—H12	109.8
C1—C2—H2	119.5	C13—C12—H12	109.8
C2—C3—C4	120.17 (12)	C14—C13—C18	120.66 (11)
C2—C3—H3	119.9	C14—C13—C12	118.67 (10)
C4—C3—H3	119.9	C18—C13—C12	120.54 (10)
C5—C4—C3	120.77 (13)	C13—C14—C15	121.45 (12)
C5—C4—H4	119.6	C13—C14—H14	119.3
C3—C4—H4	119.6	C15—C14—H14	119.3
C4—C5—C6	120.83 (13)	C16—C15—C14	116.80 (11)
C4—C5—H5	119.6	C16—C15—H15	121.6
C6—C5—H5	119.6	C14—C15—H15	121.6
C7—C6—C5	121.33 (11)	C15—C16—O4	128.34 (12)
C7—C6—C1	120.26 (10)	C15—C16—C17	121.77 (11)
C5—C6—C1	118.41 (11)	O4—C16—C17	109.88 (11)
C8—C7—C6	118.60 (10)	O3—C17—C18	127.62 (12)
C8—C7—H7	120.7	O3—C17—C16	109.94 (11)
C6—C7—H7	120.7	C18—C17—C16	122.43 (11)
C7—C8—C9	120.89 (10)	C17—C18—C13	116.89 (11)
C7—C8—C12	130.70 (10)	C17—C18—H18	121.6
C9—C8—C12	108.41 (9)	C13—C18—H18	121.6
C10—C9—C8	122.37 (11)	O3—C19—O4	108.03 (11)
C10—C9—C11	129.39 (11)	O3—C19—H19A	110.1
C8—C9—C11	108.24 (10)	O4—C19—H19A	110.1
C9—C10—C1	118.54 (11)	O3—C19—H19B	110.1
C9—C10—H10	120.7	O4—C19—H19B	110.1
C1—C10—H10	120.7	H19A—C19—H19B	108.4
			
C10—C1—C2—C3	179.83 (13)	C11—O2—C12—C8	−0.87 (12)
C6—C1—C2—C3	−0.45 (19)	C11—O2—C12—C13	120.86 (11)
C1—C2—C3—C4	0.5 (2)	C7—C8—C12—O2	−179.21 (11)
C2—C3—C4—C5	−0.3 (2)	C9—C8—C12—O2	1.19 (12)
C3—C4—C5—C6	0.0 (2)	C7—C8—C12—C13	61.35 (16)
C4—C5—C6—C7	−179.88 (12)	C9—C8—C12—C13	−118.25 (11)
C4—C5—C6—C1	−0.01 (18)	O2—C12—C13—C14	150.18 (11)
C10—C1—C6—C7	−0.20 (16)	C8—C12—C13—C14	−94.13 (13)
C2—C1—C6—C7	−179.92 (11)	O2—C12—C13—C18	−33.93 (14)
C10—C1—C6—C5	179.93 (11)	C8—C12—C13—C18	81.76 (13)
C2—C1—C6—C5	0.21 (17)	C18—C13—C14—C15	−0.74 (19)
C5—C6—C7—C8	−179.90 (11)	C12—C13—C14—C15	175.14 (11)
C1—C6—C7—C8	0.23 (16)	C13—C14—C15—C16	0.7 (2)
C6—C7—C8—C9	0.16 (16)	C14—C15—C16—O4	178.80 (13)
C6—C7—C8—C12	−179.39 (11)	C14—C15—C16—C17	−0.1 (2)
C7—C8—C9—C10	−0.61 (17)	C19—O4—C16—C15	176.89 (14)
C12—C8—C9—C10	179.03 (11)	C19—O4—C16—C17	−4.11 (15)
C7—C8—C9—C11	179.26 (10)	C19—O3—C17—C18	−176.17 (13)
C12—C8—C9—C11	−1.10 (13)	C19—O3—C17—C16	4.60 (15)
C8—C9—C10—C1	0.63 (18)	C15—C16—C17—O3	178.77 (12)
C11—C9—C10—C1	−179.21 (11)	O4—C16—C17—O3	−0.31 (15)
C2—C1—C10—C9	179.49 (12)	C15—C16—C17—C18	−0.5 (2)
C6—C1—C10—C9	−0.22 (17)	O4—C16—C17—C18	−179.59 (12)
C12—O2—C11—O1	−179.35 (12)	O3—C17—C18—C13	−178.66 (12)
C12—O2—C11—C9	0.24 (13)	C16—C17—C18—C13	0.49 (18)
C10—C9—C11—O1	0.0 (2)	C14—C13—C18—C17	0.13 (17)
C8—C9—C11—O1	−179.90 (14)	C12—C13—C18—C17	−175.68 (10)
C10—C9—C11—O2	−179.59 (12)	C17—O3—C19—O4	−7.07 (15)
C8—C9—C11—O2	0.56 (13)	C16—O4—C19—O3	6.88 (15)

### Hydrogen-bond geometry (Å, °)

**Table:**

Cg1 is the centroid of the C13–C18 ring.

**Table:**

*D*—H···*A*	*D*—H	H···*A*	*D*···*A*	*D*—H···*A*
C12—H12···O1^i^	0.98	2.39	3.3277 (16)	159
C3—H3···Cg1^ii^	0.93	2.84	3.6021 (15)	140

Symmetry codes: (i) −*x*+1, *y*−1/2, −*z*+1/2; (ii) −*x*+2, −*y*+2, −*z*.

## References

[bb1] Bruker (2004). *APEX2* and *SAINT* Bruker AXS Inc., Madison, Wisconsin, USA.

[bb2] Farrugia, L. J. (1997). *J. Appl. Cryst.* **30**, 565.

[bb3] Howlett, D. R., Perry, A. E., Godfrey, F., Swatton, J. E., Jennings, K. H., Spitzfaden, C., Wadsworth, H., Wood, S. J. & Markwell, R. E. (1999). *Biochem. J.* **340**, 283–289. PMC1220247 10229684

[bb4] Sheldrick, G. M. (1996). *SADABS* University of Gottingen, Germany.

[bb5] Sheldrick, G. M. (2008). *Acta Cryst.* A**64**, 112–122. 10.1107/S0108767307043930 18156677

[bb6] Spek, A. L. (2009). *Acta Cryst.* D**65**, 148–155. 10.1107/S090744490804362X PMC2631630 19171970

[bb7] Twyman, L. J. & Allsop, D. (1999). *Tetrahedron Lett.* **40**, 9383–9384.

[bb8] Valerga, P., Puerta, M. C., Rodríguez Negrín, Z., Castañedo Cancio, N. & Palma Lovillo, M. (2009). *Acta Cryst.* E**65**, o1979. 10.1107/S160053680902861X PMC2977326 21583655

